# Inhibition of current perception thresholds in A-delta and C fibers through somatosensory stimulation of the body surface

**DOI:** 10.1038/s41598-022-18016-y

**Published:** 2022-08-12

**Authors:** Kazuhiro Shimo, Sho Ogawa, Yuto Niwa, Yuji Tokiwa, Ayaka Dokita, Sho Kato, Takafumi Hattori, Takako Matsubara

**Affiliations:** 1 Department of Physical Therapy, Faculty of Rehabilitation, Kobe Gakuin University, 518 Arise, Ikawadani-cho, Nishi-ku, Kobe, Hyogo 651-2180 Japan; 2 Faculty of Rehabilitation, Kobe Gakuin University Graduate School, Kobe, Japan; 3 Department of Rehabilitation, Amagasaki Daimotsu Hospital, Amagasaki, Japan; 4 Department of Rehabilitation, Maehara Orthopedics Rehabilitation Clinic, Obu, Japan

**Keywords:** Sensory processing, Somatosensory system

## Abstract

Somatosensory stimulation of the body surface, such as through tactile and noxious stimulation, is widely known to inhibit pain. However, no studies have measured the threshold changes due to somatosensory stimulation of each nerve fiber (Aβ, Aδ, and C) separately. We examined the changes in the current perception thresholds of Aδ, C, and Aβ fibers induced by non-noxious and noxious somatosensory stimulation of the body surface. The current stimuli were sinusoidal waves at frequencies of 2000 Hz, 250 Hz, and 5 Hz, which selectively stimulated the Aβ, Aδ, and C fibers, respectively. In the case of non-noxious stimulation, lightly rubbing the dorsal side of the forearm with a brush showed no significant physiological or clinical changes in the current perception thresholds of the Aδ, and C fibers; a significant increase was observed only in the Aβ fibers. However, applying noxious stimulation to the body surface through hand immersion in cold water increased pain thresholds in both the Aδ and C fibers, and sensory threshold of the Aβ fibers; changes in tactile thresholds were not significant. Inhibition of sensory information by nociceptive inputs may selectively suppress nociceptive stimuli.

## Introduction

Pain relief is often achieved by rubbing or pressing the painful area. Apart from tactile stimulation, noxious stimulation is also known to provide pain relief. As such, somatosensory stimulation of the body surface is widely known to inhibit pain. However, the mechanism underlying pain inhibition through somatosensory stimulation of the body surface remains unclear^1^.

The gate-control theory is often used to explain the mechanism of pain inhibition using non-noxious stimuli, such as rubbing the affected area or application of transcutaneous electrical nerve stimulation. The gate-control theory, published by Melzack and Wall in 1965^2^, states that the dorsal horn of the spinal cord has a gate function that controls the input of nociceptive information. Activation of Aβ fibers, which transmit tactile stimuli, increases the excitability of inhibitory interneurons, and nociceptive information from Aδ and C fibers is inhibited through presynaptic inhibition. Although the gate-control theory is a good model for explaining the pain-inhibitory response to stimulation of the body surface, it has not been scientifically proven.

Diffuse noxious inhibitory controls (DNIC)^3,4^ and conditioned pain modulation (CPM)^5^ refers to pain inhibition in other regions of the body through stimulation of the body surface. The DNIC/CPM is considered to reflect the function of the descending pain modulatory system^6^. The descending pain modulatory system comprises a network of cortical and subcortical brain regions that can inhibit nociceptive afferent brain input via brainstem structures like the periaqueductal gray matter and the rostral ventromedial medulla. However, whether nociceptive stimuli specifically inhibit nociceptive information has not been sufficiently investigated.

Most previous studies on pain inhibition through somatosensory stimulation have used subjective pain intensity changes as the outcome. This may explain why the mechanism of pain inhibition through somatosensory stimulation in humans remains unclear. In recent years, studies using quantitative sensory testing (QST) to assess pain have been conducted^7^. The QST is a psychophysical assessment that evaluates sensory responses to standardized stimuli. The stimuli used in QST include mechanical (pressure, pinprick, vibration), temperature (heat and cold), and electric current stimuli^8,9^. Furthermore, the responses of Aδ and C fibers in nociceptive neurons and Aβ fibers in non-nociceptive neurons have been evaluated selectively through transcutaneous electrical nerve stimulation at different frequencies. Assessment of quantitative current perception thresholds (CPT) has been reported to be useful for evaluating sensory function. Sinusoidal electrical currents of 2000, 250, and 5 Hz have been observed to stimulate Aβ, Aδ, and C fibers, respectively^10^. The electrical stimulus produced by the Neurometer®^10^ device is self-calibrating and maintains a constant current output regardless of normal variations in skin thickness and impedance. The system monitors the impedance at the skin electrode interface that could distort the accuracy of the measures. The reproducibility of Neurometer® has been shown by Furuse et al.^11^.

In this study, we examined the changes in CPT of the Aδ, Aβ, and C fibers induced by somatosensory stimulation of the body surface, respectively. Little is known about the threshold changes due to somatosensory stimulation of each nerve fiber (Aβ, Aδ, and C) separately. We aimed to determine the effects of somatosensory stimulation on pain and tactile perception in humans.

## Results

**Figure 1:**
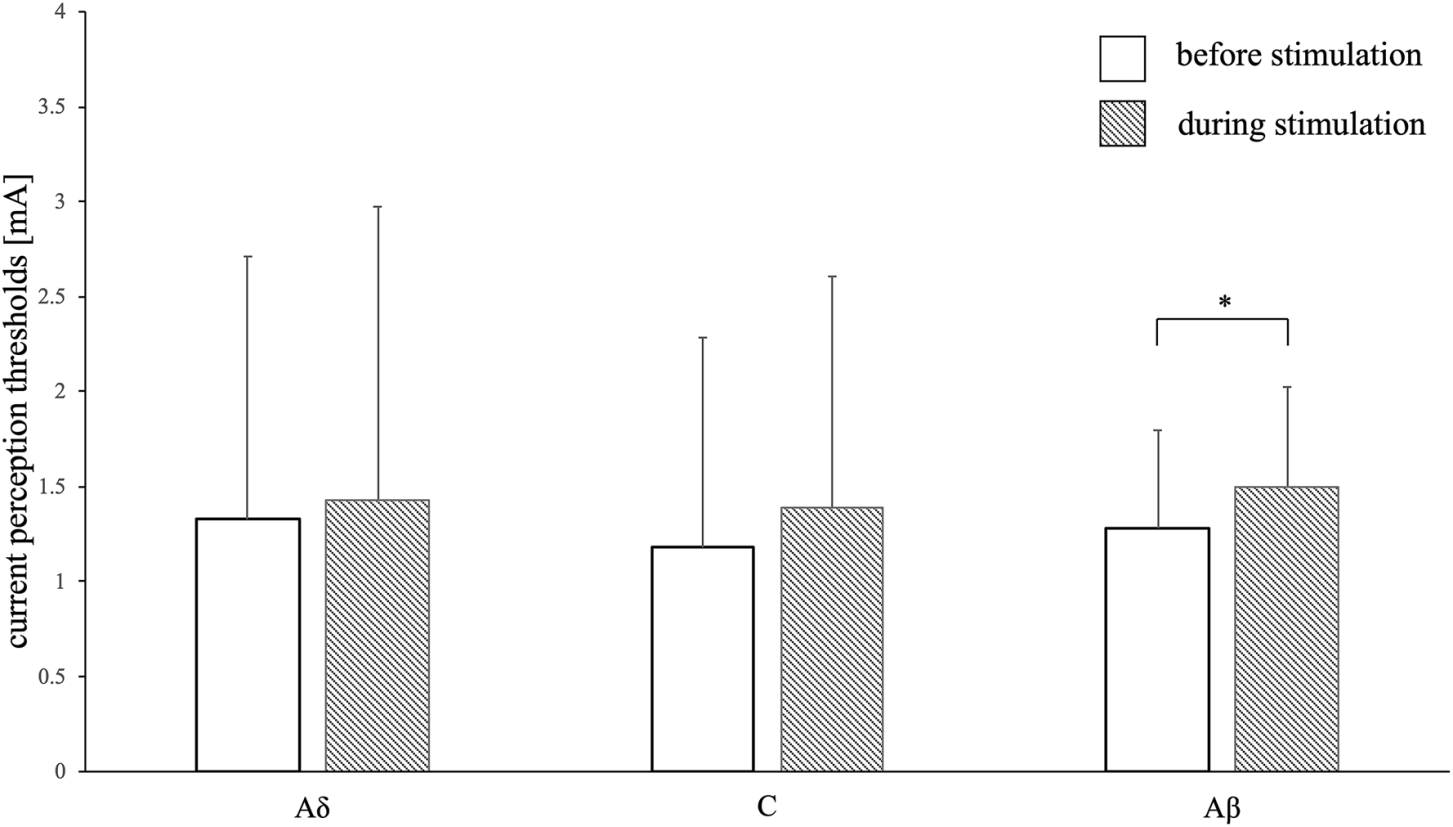
Current perception thresholds under non-noxious condition. Differences in the mean value of current perception thresholds before and after non-noxious stimulation. The error bars indicate standard deviation. No significant changes are observed in the current perception thresholds of the Aδ and C fibers when the dorsal side of the forearm was lightly rubbed with a brush. A significant increase in the threshold of the Aβ fibers is observed. Asterisks represent significant differences (*P < 0.0083).

**Figure 2:**
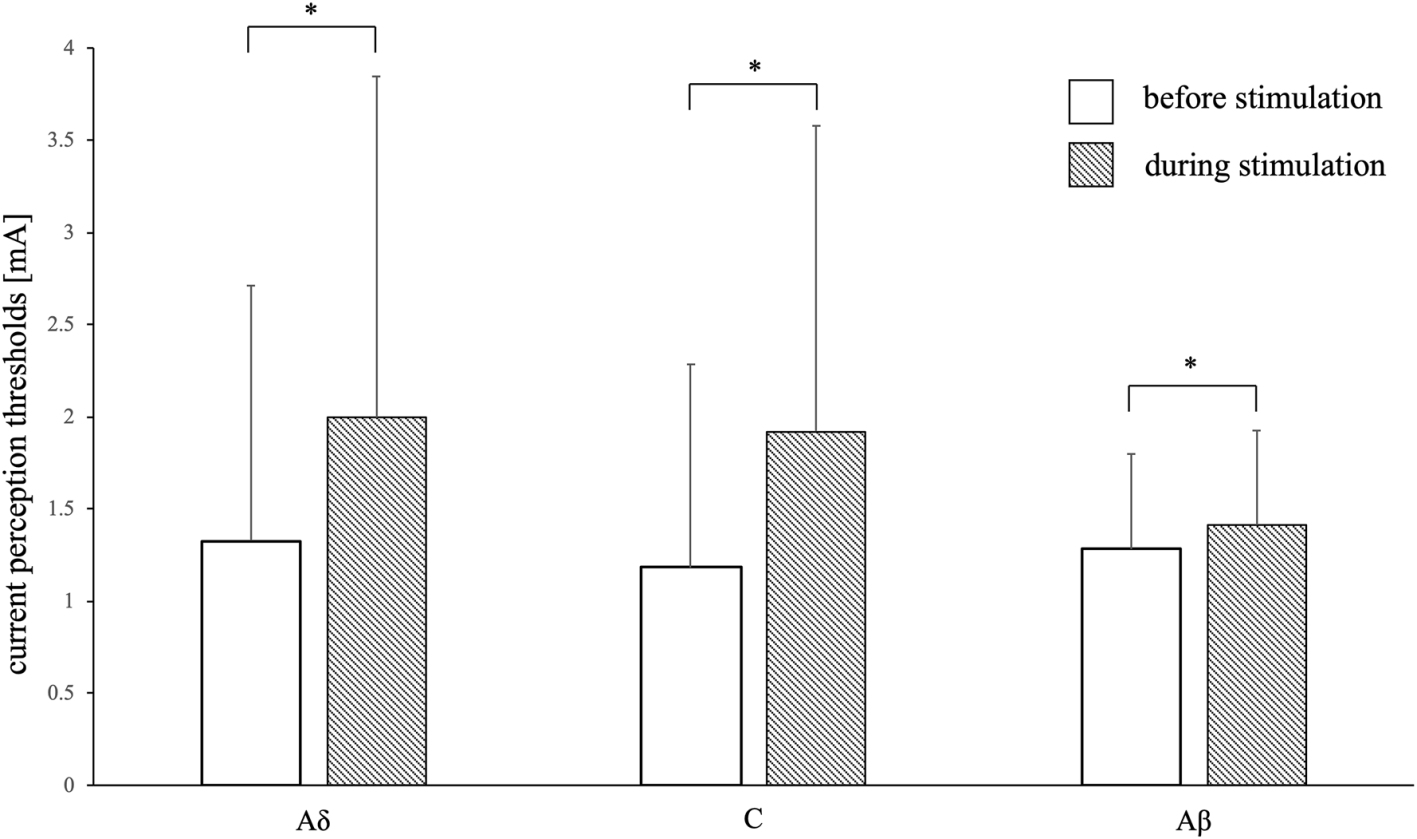
Current perception thresholds under noxious condition. Differences in the mean value of current perception thresholds before and after noxious stimulation. The error bars indicate standard deviation. Significant increases are observed in the current perception threshold of the Aδ, C, and Aβ fibers after hand immersion in cold water. Asterisks represent significant differences (*P < 0.0083).

Before body surface stimulation, the CPTs were 1.33 ± 1.38 mA, 1.18 ± 1.10 mA, and 1.28 ± 0.52 mA for the Aδ, C, and Aβ fibers, respectively. During body surface stimulation, the CPTs were 1.43 ± 1.54 mA, 1.39 ± 1.22 mA, and 1.50 ± 0.52 mA for the Aδ, C, and Aβ fibers, respectively, in the non-noxious condition, and 1.99 ± 1.85 mA, 1.92 ± 1.66 mA, and 1.41 ± 0.51 mA for the Aδ, C, Aβ fibers, respectively, in the noxious condition. The CPTs were significantly increased in the Aβ fibers (*P* = 0.0000604) under non-noxious conditions and in the Aδ (*P* = 0.000181), C (*P* = 0.00000447), and Aβ (*P* = 0.000798) fibers under noxious conditions (Figs. 1 and 2).

## Discussion

In this study, we examined the changes in the CPTs of Aδ, C, and Aβ fibers when non-noxious or noxious stimulation was applied to the body surface of healthy participants. In the case of non-noxious stimulation, lightly rubbing the dorsal side of the forearm with a brush showed no significant changes in the CPT of the Aδ and C fibers and a significant increase was observed only in the Aβ fibers. In contrast, noxious stimulation through hand immersion in cold water significantly increased all CPTs of Aδ, C and Aβ fibers. However, the mean increase in the CPT of Aβ fibers was 0.22 mA under non-noxious conditions and 0.13 mA under noxious conditions. Previous studies have reported that the difference in CPT between pre- and post-treatment and with and without peripheral neuropathy is approximately 0.5 mA^12–14^. Therefore, the increase in the CPT of the Aβ fibers in this study was minor under both non-noxious and noxious conditions. Although there was a statistically significant difference, it seemed to have little physiological or clinical significance. However, the previous study was conducted on individuals who were ill and had decreased tactile perception, and the study examined changes over a long period rather than a single intervention that may have resulted in a larger change in thresholds than the present results.

Pain inhibition through non-noxious stimulation of the body surface is often experienced as touch-induced analgesia^15^, in which pain is relieved by rubbing the painful area. However, in this study, no significant change in pain threshold was observed in either the Aδ or C fibers, despite light stimulation through rubbing with a brush. Moreover, as mentioned above, the changes in the sensory thresholds of the Aβ fibers were minor and of little physiological or clinical significance. The following two factors might have influenced the results of this study. First, the pain threshold obtained through current stimulation was used as an index of pain inhibition rather than subjective pain intensity; second, stimulation through light rubbing with a brush was chosen as the non-nociceptive stimulation method. The pain threshold was defined as the current intensity at which the participant first became aware of the stimuli when the current intensity of the Aδ or C fibers gradually increased. Therefore, the subjective pain intensity in this study was extremely low, and there might have been a discrepancy in the findings due to touch-induced analgesia experienced in daily life. Previous studies^14,16,17^ have evaluated changes in subjective pain intensity as an index of pain inhibition through light tactile stimulation. Additionally, the gate-control theory has been used to define the mechanism of pain inhibition through non-noxious stimulation, such as tactile stimulation of the body surface. According to the gate-control theory, the thick fibers activate inhibitory interneurons in layer II of the dorsal horn of the spinal cord, called the substantia gelatinosa, and inhibit the transmission of nociceptive information by the thin fibers. In this study, a light-rubbing stimulus with a brush was used. Pleasant tactile stimuli, such as the one mentioned above, are generally transmitted not only by Aβ fibers but also by C fibers, which transmit tactile sensations^17–19^. As per the gate-control theory, the thin nerve fibers that transmit nociceptive information suppress inhibitory interneurons in the dorsal horn of the spinal cord; that is, they have the effect of opening the gate. Although the light rubbing stimulus used in this study was a tactile stimulus, it acted on opening the gate by inhibiting the inhibitory interneurons in the dorsal horn of the spinal cord through the transmission of stimulus information by the thin C fibers, which might have prevented the expected gate-control effect on pain.

In contrast, the nociceptive stimulus provided through immersing the hand in cold water increased the pain threshold of both the Aδ and C fibers. This change in the pain threshold of the Aδ and C fibers can be regarded as a sufficient amount of change compared to those observed in previous studies^12–14^. In contrast, the change in the perceptual threshold of the Aβ fibers in this study was not significant or sufficient, as described above. Our results indicate that noxious stimulation of the body surface inhibits relay of nociceptive information in other parts of the body. The inhibition is limited to input of nociceptive information from the Aδ and C fibers and does not affect information from the Aβ fibers. These results suggest that nociceptive stimuli may selectively inhibit pain. Pain inhibition by noxious stimulation, such as immersion in cold water, is also considered a distraction from other somatosensory information since noxious stimulation serves as critical warning signals. This corresponds with the findings of a previous study^6^ that the DNIC/CPM, in which pain is inhibited by noxious stimulation of other parts of the body, reflects the function of the descending pain modulatory system, suggesting that noxious stimulation of the body surface triggers the central pain control mechanism.

In this study, we investigated the pain-inhibitory response to somatosensory stimulation of the body surface through QST using current stimuli applied to different peripheral nerve fibers. Current stimuli have the advantages of easy control, such as changing the stimuli intensity or switching it on/off, high reproducibility, and the possibility of setting up various stimulation protocols. Conventionally, thermal laser stimulation has been used to selectively stimulate the Aδ and C fibers, which allows nerve evaluation without the influence of the Aβ fibers; this is because stimulation can be performed without contacting the skin with the stimulator^20,21^. However, thermal laser stimulators are an expensive equipment and require complex clinical neurophysiological tests. The Neurometer® CPT/C used in this study can selectively and easily stimulate peripheral nerves according to fiber type by setting the frequency of current stimulation. The QST of peripheral nerves according to fiber type has rarely been investigated, and it is expected that the method used in this study will be applied in future studies to explore the pain inhibition mechanism in humans, and further developed to evaluate pain regulatory functions. The CPT was used as an index of pain sensitivity. However, to examine pain inhibition responses in detail, it is necessary to evaluate pain tolerance values and subjective pain intensity in response to current stimulation at a specific intensity. For pain inhibition through non-noxious stimulation, in particular, setting up an experimental protocol that is more in line with daily experiences will clarify the mechanism.

In this study, we were not able to set up controls to directly compare the effects of attention. When stimulation of other sites reduces attention to the main stimulus site, it is expected that the effects on each nerve fiber would not be different, but would affect them equally. However, the fact that the effects of stimulation of other sites differed by nerve fiber type in this study suggests that not only simple attention but also sensory input influenced nerve fibers. However, since the influence of attention to other sites on sensory thresholds is expected to be significant, experimental protocols should be considered in the future that eliminate this influence. The non-nociceptive stimulus used in this study was a light touch, but the pressure was not measured and the details are unknown. The light touch is assumed to be milder than a stimulus such as rubbing the pain site. In addition, there is no concern about adverse effects on the human body regarding the suppression of neurotransmission by non-nociceptive stimulation, even if the measurement time is prolonged, so future studies should be conducted using the original forced-choice tests.

In conclusion, applying noxious stimulation to the body surface increased pain thresholds of both the Aδ and C fibers, whereas changes in tactile thresholds were not significant. Inhibition of sensory information by nociceptive inputs may selectively suppress nociceptive stimuli.

## Methods

### Participants

All participants were healthy, right-handed volunteers (n = 30; 20 women; mean age, 22.5 ± 2.3 years; average body mass index, 20.9 ± 1.9).

Ethics approval was obtained from the Institutional Ethics Committee of Kobe Gakuin University in Kobe, Japan (No.: So-Rin 19–22). Written informed consent was obtained from all participants before the study. This study was conducted in compliance with the Declaration of Helsinki and its subsequent amendments.

### Body surface somatosensory stimulation

Body surface stimulation was performed under non-noxious and noxious conditions. In the non-noxious condition, stimulation consisted of slow brush-stroking at a velocity of ~ 3 cm/s and an approximate indentation force of 0.3 N^16^ in the proximal to distal direction on the dorsal forearm of the dominant hand. All stroking stimuli were delivered manually by an experimenter trained to apply the strokes with constant force and velocity. In the noxious condition, participants immersed their non-dominant hands into a circulating water bath maintained at a temperature of 6 − 8 °C^22–24^. In reference to a previous study^25^, an interval of 20 min was allowed between stimuli.

### Current perception thresholds

We measured the CPT using a Neurometer® CPT/C (Neurotoron Inc., Baltimore, MD) to evaluate the sensory response of the peripheral nerves. The dorsal side of the dominant forearm (distal to the second lateral finger from the lateral epicondyle of the humerus on the line connecting the radial stalk and lateral epicondyle of the humerus) was used as the measurement site. The current stimuli were sinusoidal waves at frequencies of 2000 Hz, 250 Hz, and 5 Hz, which selectively stimulated the Aβ, Aδ, and C fibers, respectively. In the current stimuli, the current gradually increased from 0 mA. The stimulation would automatically stop if the maximum intensity (9.99 mA) was reached. The duration of each test step was a function of the stimulus frequency: 0.72 s at 2000 Hz (20 steps), 2.16 s at 250 Hz (20 steps), and 2.52 s at 5 Hz (29 steps). When the participant felt the stimulation for the first time, they used their dominant hand to press the stop button and the current at that time was measured and set as the current threshold. The time to press the button includes the time between perception and the onset of motion. The Neurometer® CPT/C compensates for this lag by assuming that the button is pressed in response immediately after perception. The time difference between the participant’s perceptions of the stimulation and the pressing of the stop button was automatically corrected by the device.

Measurements were performed before and during the somatosensory stimulation of the body surface. The order of the measurement of the two body surface stimulation conditions and the three frequencies of the current stimulation were random. All subjects were thoroughly practiced in measuring the CPT prior to the experiment. Only one CPT measurement in the experiment was made per condition and per frequency.

### Statistical analysis

The means and standard deviations of the data were calculated and presented. Wilcoxon’s signed-rank test was used to examine the changes in sensory thresholds due to surface stimulation. We calculated the Bonferroni-adjusted significance level and considered a p-value of < 0.0083 to be significant to account for the increased possibility of a type-1 error (α = 0.05).

## Data Availability

The datasets generated and/or analyzed during the current study are available from the corresponding author upon reasonable request.
